# Engendering health systems in response to national rollout of dolutegravir-based regimens among women of childbearing potential: a qualitative study with stakeholders in South Africa and Uganda

**DOI:** 10.1186/s12913-020-05580-0

**Published:** 2020-08-01

**Authors:** Yussif Alhassan, Adelline Twimukye, Thoko Malaba, Catherine Orrell, Landon Myer, Catriona Waitt, Mohammed Lamorde, Andrew Kambugu, Helen Reynolds, Saye Khoo, Miriam Taegtmeyer

**Affiliations:** 1 Community Health Systems Group, Liverpool School of Tropical Medicine, Pembroke Place, Liverpool, L3 5QA UK; 2 Infectious Disease Institute, Kampala, Uganda; 3 School of Public Health and Family Medicine, University of Cape Town, Cape Town, South Africa; 4 Desmond Tutu HIV Centre, University of Cape Town, Cape Town, South Africa; 5 Institute of Translational Medicine, University of Liverpool, Liverpool, UK

**Keywords:** Health system strengthening, Dolutegravir-based regimen, Women of childbearing potential, Qualitative study, Uganda, South Africa

## Abstract

**Background:**

In the era of rapid dolutegravir rollout, concerns about neural tube defects have complicated the health systems response among women of childbearing potential. This qualitative study, which was nested within the DolPHIN-2 clinical trial, examined the current and future health system opportunities and challenges associated with the transition to dolutegravir-based regimen as first line antiretroviral therapy among women of childbearing potential in South Africa and Uganda*.*

**Method:**

Semi-structured in-depth interviews with members of antiretroviral therapy guideline development groups and affiliates were conducted. Thirty-one participants were purposively selected for the study, including senior officials from the Ministry of Health and National Drug Regulatory Authority in Uganda and South Africa as well as health-sector development partners, activists, researchers and health workers. A thematic approach was used to analyse the data.

**Findings:**

Despite differences in health system contexts, several common challenges and opportunities were identified with the transition among women of childbearing potential in South Africa and Uganda. In both contexts national stakeholders identified challenges with ensuring gender equity in roll out due to the potential teratogenicity of dolutegravir, paucity of data on dolutegravir use in pregnancy, potential stock out of effective contraceptives, poorly integrated contraception services, and limited pharmacovigilance in pregnancy. Participants identified opportunities that could be harnessed to accelerate the transition, including high stakeholder interest and commitment to transition, national approval and licensure of a generic tenofovir/lamivudine/dolutegravir regimen, availability of a network of antiretroviral therapy providers, and strong desire among women for newer and more tolerable regimens.

**Conclusion:**

The transition to dolutegravir-based regimens has the potential to strengthen health systems in low- and middle-income countries to engender equitable access to optimised antiretroviral regimen among women. There is the need for a multi-sectoral effort to harness the opportunities of the health systems to addresses the bottlenecks to the transition and initiate extensive community engagement alongside individual and institutional capacity strengthening. Improvements in pregnancy pharmacovigilance and counselling and family planning services are critical to ensuring a successful transition among women of childbearing potential.

## Background

Since 2016 there has been a concerted effort to implement dolutegravir-based first line antiretroviral therapy regimens in low- and middle-income countries (LMICs) following the World Health Organisation (WHO) guidelines releases in 2016 [1] and 2018 [2]. The impetus for transition to dolutegravir reflects its advantages over current first line efavirenz-based regimens, including improved viral suppression and tolerability as well as a higher genetic barrier to resistance [3, 4]. Programmatically, large-scale rollout of dolutegravir has potential to increase harmonisation, simplify drug procurement and lower costs [2]. Recent modelling studies indicate overall public health benefits from wider dolutegravir use [5, 6] despite its potential association with neural tube defects when used periconception [7].

After initial enthusiasm a more cautious approach to dolutegravir use among women of childbearing potential was recommended in 2018. This included avoiding use in the periconception period, ensuring reliable contraception and informed choice [2]. Use in second and third trimesters of pregnancy was not contraindicated. In July 2019, WHO downgraded the risk of neural tube defects following new evidence and recommended dolutegravir as a preferred regimen for women of childbearing potential, although the caution remains [8].

Many LMICs have either adopted or planning to switch to dolutegravir-based regimens for first line treatment [9]. It is estimated that by 2021 approximately 15 million people would be using dolutegravir-based regimens, with non-nucleoside reverse-transcriptase inhibitor-based regimens being rapidly replaced [10]. South Africa and Uganda have revised their ART guidelines to transition to dolutegravir-based first-line HIV regimens and away from efavirenz-containing regimens. These countries have high rates of HIV prevalence (South Africa 20.4% and Uganda 5.7%), people on antiretroviral treatment (South Africa 4.4million; Uganda 940,000) [11], and pre-treatment resistance to non-nucleoside reverse-transcriptase inhibitor-based regimens (Uganda 15.9% and South Africa 23.6%) [12]. In Uganda, national roll-out of dolutegravir-based regimens commenced in March 2018 with access initially restricted among women of childbearing potential but later extended to include all adults in line with new WHO guidelines [13]. In South African guidelines published in October 2019, dolutegravir is recommended as the preferred regimen for all adults except in women around the time of conception and in the first trimester of pregnancy. Women of childbearing potential are encouraged to use contraception if taking dolutegravir [14].

Experience from previous transition in ART regimens suggests that the transition process can place profound strain on the health system, particularly in resource-limited countries. With health systems in LMICs under-resourced, reliant on donor-funded vertical programmes, and care for human immunodeficiency viruses (HIV) delivered by lower level cadres through standardised protocols, implementing the nuanced WHO approach to the transition among women of childbearing potential could pose significant challenges. In this study, we explored the potential health system opportunities and challenges to transitioning to dolutegravir-based first line regimen among women of childbearing potential in South Africa and Uganda. We aimed to draw lessons that would resonate with other LMICs as they prepare to change their first line regimen to dolutegravir-based regimen.

## Methods

### Study design

This study forms part of the qualitative component of the DolPHIN-2 trial (NCT03249181) which aims to evaluate the efficacy and safety of dolutegravir use in pregnancy. Early results from the trial indicate that dolutegravir is well-tolerated and achieves superior virological suppression compared to efavirenz when initiated in late pregnancy [15]. The current study was set in South Africa and Uganda because they are study sites for the DolPHIN-2 trial and provide suitable contexts for understanding LMICs health systems.

We employed a qualitative exploratory design in this study to better understand stakeholders perspectives of the health systems opportunities and challenges to the transition and the context in which those perspectives are situated [16]. In order to obtain a holistic view of the health system, we used the WHO health system building blocks framework to determine potential areas of impact by the transition to focus our investigation [17, 18]. The approach conceptualises the health system as encompassing six functional components including stewardship, financing, information systems, human resources, service delivery and medical products. It also underscores the role of communities (people) as mediators and beneficiaries of the functions of the health system as well as equity, efficiency, responsiveness and financial protection as important health system goals [19]. We focused mainly on the six functional components of the health system plus the community to ascertain the potential opportunities and challenges to the transition.

### Participant selection

Purposive sampling was applied to select respondents. Given the complex and prospective nature of transitioning (rollout had not started during the study), we sought to understand the potential issues through the perspectives of key informants [20]. Key informant accounts provide a generalised and summarised view of health care system transition and are informed by previous and current experience as well as future expectation, which are useful for prospective studies [21]. Participants had several years of working in their fields (Table 1) and were mainly members of the ART guidelines technical working group (TWG) and affiliates in South Africa and Uganda. They included Ministry of Health (MOH) officials involved in HIV policy and programmes, clinicians, researchers, activists, HIV development partners, and national drug regulators (Table 1). The researchers and MOH officials who were selected were all members of the guidelines TWG in their respective countries; the clinicians in Uganda were involved in the implementation of a transition pilot; the regulators were responsible for licensing and monitoring the safety of dolutegravir; and the development partners provided funding and technical assistance to the transition. We identified key institutions known to be involved in the transition and invited senior officials who were directly involved in the process to participate. Snowball sampling was used to supplement the initial participant list. Participants were recruited and interviewed until no new themes emerged and saturation was reached [22]. A total of 31 key informants participated in the study across the two study settings.

**Table 1:** Background characteristics of study participants^a^

	South Africa n (16)	Uganda n (15)	Total n (31)
Sector			
Ministry of Health	2	2	4
Regulator	4	1	5
Researcher	7	3	10
Activist	2	3	5
Clinician	0	3	3
Development partner	1	3	4
Sex			
Male	10	3	13
Female	6	12	18
Number of years working in field			Range: 5–35 Median: 15.6
Age			≥ 25 years

^a^Some participant categories overlap due to multiple roles

### Data collection

Data collection was conducted between January and August 2018, at the time of participant recruitment for the DolPHIN-2 trial. The period coincided with the releases of preliminary results from the Tsepamo study and the WHO safety alert on the potential association of dolutegravir use in periconception with risk of neural tube defects [23, 24], which may have shaped participants’ responses. Neither study countries had started rolling out dolutegravir-based regimens at the time of the interviews; and were in the process of revising guidelines and preparing for transition. Semi-structured topic guides were used to explore participants’ perceptions about current and anticipated challenges and opportunities and how experiences from previous ART rollouts may inform the current transition. The topic guides initially developed by YA and MT were based on the WHO 2018 ART guidelines [2] and the WHO building block framework. They focused on key policy and programmatic issues in relation to the transition, including guidelines development/revision, commodity procurement and supply, delivery of dolutegravir based treatment, health worker capacity, pharmacovigilance, and community acceptability. The topic guides were piloted and revised iteratively as the interviews evolved. All interviews were conducted in English, either face-to-face or telephonically, by experienced senior qualitative researchers (YA, MT, AT and TM), and audio recorded. The interviews were complemented by a review of literature and policy documents to provide background information to place our findings into context. These included ART guidelines, the South Africa National Strategic Plan for HIV, TB and STIs (2017–2022), the Uganda National HIV and AIDS Stratergic Plan (2015/2016–2019/2020), and the Uganda Presidential Fast Track Action plan.

### Data analysis

**Fig. 1:**
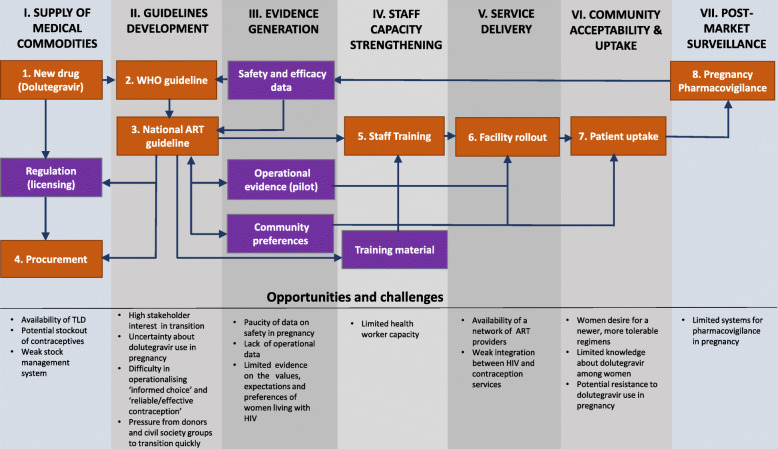
Potential health system opportunities and challenges to transition to dolutegravir-based regimens. Diagram outlines key elements of the health system involved in transitioning to dolutegravir-based first line antiretroviral regimens among women of childbearing potential, and related potential opportunities and challenges identified in South Africa and Uganda*.* Orange rectangle shapes represent major activities of the transition process and the purple shapes represent auxiliary activities/elements. Arrows denote relationship between the transition elements/activities

Audio-recorded interviews were transcribed verbatim, with each being reviewed for completeness and accuracy. Data were analysed in NVivo 11 software based on a thematic approach. Themes were inductively generated based on ideas that emerged from the data [25]. Each transcript was read and reread by YA, AT and MT for recurrent ideas. Codes were assigned to relevant segments of the text; similar codes were aggregated to form themes that were then used to address the research questions and develop coherent narratives [26]. Identified themes were categorised under key components of the transition process (see Fig. 1). Contradictory data identified during the analysis were initially treated as potential different viewpoints; they were subjected to further analyses to validate or refute them; validated data were used to enrich the perspectives on the issue [27]. To ensure rigor and trustworthiness, transcripts were independently coded, compared and discussed, and a common coding framework was used [25]. Inter-rater agreement between the analysts was moderate (Kappa coefficient = .75), discrepancies were discussed and resolved by consensus at coding meetings [28]. Emerging findings from the analysis were discussed among the authors in regular qualitative research network meetings, and reponsdent validation was ensured by presenting draft results to the stakeholders with subsequent feedback integrated into the analysis.

### Ethical considerations

Ethical approval for the study was obtained from ethics review commitees of the Liverpool School of Tropical Medicine (LSTM REC 17–087), the University of Cape Town Faculty of Health Sciences (HREC REF: 280/2018) and the Joint Clinical Reseach Centre in Uganda (JC0818). Participation in the study was voluntary and written and/or verbal informed consent was obtained from participants before data collection. All participant personal information and identity were removed during analysis and reporting, and privacy and annonymity were maintained by applying alphanumeric codes. All data were password protected and stored on encrypted computers and locked cabinets.

## Results

Despite difference in health system context, participants identified several common opportunities and challenges to the transition to dolutegravir-based regimens among women of childbearing potential in South Africa and Uganda. Whilst the WHO building blocks were used to structure our enquiry, we found that some of the themes that emerged from the data were not clearly aligned with the categories of the health system components and some aspects of the health system components were more critical than others for the transition. Therefore, we presented the findings in line with Fig. 1, which outlines key components of the transition process and related opportunities and challenges, enabling us to better highlight where the WHO building blocks are particularly affected in the transition. The key components of the transition process include guidelines development, evidence generation, supply of medical commodities, staff capacity and service delivery, community acceptability, and uptake and post-market surveillance.

### Guidelines development

#### Stakeholder interest in transition to dolutegravir among women

Nearly all the participants agreed that it was essential to switch to dolutegravir-based regimens due to the numerous potential benefits that it provided, especially for women. They noted high interest and commitment to the transition among politicians, donors, civil society groups and providers in both countries. Several of the MOH staff stated that they were motived by dolutegravir’s high genetic barrier to resistance. Potential cost savings associated with widespread use of dolutegravir-based regimens was particularly attractive to both national policymakers and donors. Civil society actors were driven by the potential direct health benefits of dolutegravir to women, especially low side effects, rapid viral suppression, and reduced risk of vertical transmission. Despite existing health system challenges, most participants were optimistic that their countries could successfully transition to dolutegravir-based regimens (including amongst women) due to the high stakeholder interest and experience from previous ART regimens changes.

*“Everybody is committed. … they all want to be part and are very supportive because of its [ dolutegravir] benefits. … Because of its potential to reduce vertical transmission there is an interest to ensure that women are able to access it. I’m very sure transition is possible. This is not going to be the first time that we have change HIV drugs … . There will definitely be challenges but together I’m sure we can overcome them.”* (Uganda, MOH staff, P12)

#### Uncertainty about dolutegravir use in pregnancy

A major challenge to developing ART guidelines for the transition in both countries was uncertainty about dolutegravir use in pregnancy (and among women of childbearing potential) due to potential association with neural tube defects. There was reported long delay in revising ART guidelines due to disagreement over how to balance the public health risks and individual benefits of dolutegravir use among women of childbearing potential. Several guidelines TWG members noted that the debate presented ethical conundrums which they lacked adequate information to deal with.

*“How to balance the risks [of* neural tube defects *to the child] and benefits [to the mother] of DTG [dolutegravir] is a source of worry and delaying us. … it raises ethical issues which we are not well equipped to handle”* (South Africa, Researcher, P2)

#### Difficulty in operationalising ‘informed choice’ and ‘reliable/effective contraception’

While welcoming the WHO recommendation for informed choice over dolutegravir use among women of childbearing potential, several guidelines TWG members across both countries reported difficulties in operationalising ‘informed choice’ citing how hard it was to define ‘choice’ and ‘informed’, and how to document informed choice. They also expressed ambiguity over the WHO term ‘reliable/effective contraception’ and stressed the need for greater clarity on the term and informed by community perspectives.

*“What they [WHO] say ‘reliable method of contraception’? what does it mean in practice? Is it someone saying that they are not sleeping with anyone? Are condoms a reliable method of contraception? is it injectables? it is hormonal contraception? So, I think in the end the understanding of these loaded words from a community perspective is important”* (South Africa, Researcher, P6).

#### Pressure to transition quickly

Some MOH staff lamented over pressure from several donors and patient advocacy organisations to transition quickly and allow dolutegravir use among women of childbearing potential even when the health systems were not prepared and lacked sufficient evidence on the safety of dolutegravir use in pregnancy.

*“Unfortunately, we can’t wait [for the evidence on the safety of dolutegravir] even when we are limited by that because there is a lot of pressure from civil society as well as PEPFAR. PEPFAR has given us a fair amount of money to move towards a dolutegravir-based [regimen].”* (South Africa, MOH staff, P1)

### Evidence generation

#### Paucity of evidence on dolutegravir use in pregnancy

Lack of data on the safety of dolutegravir in pregnancy was identified as a major barrier to transition among women. In South Africa, insufficient safety data in pregnancy was widely mentioned as a key reason for the delay in the national rollout. Several guidelines TWG members identified the Tsepamo study as their main source of safety data on dolutegravir but noted it was inadequate due to being an observational study. Participants expressed the need for more clinical trial data.

“ *… neural tube defect is making life difficult for us as we develop the guidelines. It is difficult to make a decision on how to roll it out among women. We only have the Botswana data to work with but that one has its challenges. We need more trial data. … . it should cover all aspects of pregnancy.”* (South Africa, MOH staff, P7)

Further, participants identified a lack of community and operational evidence to inform the transition. Unlike Uganda, no pilot study had been conducted in South Africa to support rollout, leading to guidelines TWG members calling for data on the expectations, values and preferences of women living with HIV on dolutegravir treatment. Whilst both countries had previously implemented ART transition (e.g. replacement of stavudine and the change from nevirapine to efavirenz), there was also a sense that the current transition with its emphasis on nuanced treatment services among women (due to the potential teratogenicity of dolutegravir) was different and needed to be informed by more dolutegravir-specific data. Participants across both countries believed that evidence generation was being hindered by the rapid pace of the transition.

*“We are likely to face problems, just like we have done in the past [transition] because we haven’t taken our time to consult with those who will be affected. … there is pressure to get it [transition] started as soon as possible.”* (Uganda, MOH staff, P5).

### Supply of medical commodities

#### Availability of TLD

Participants were optimistic about adequate supply of dolutegravir-based regimen during the transition. Both countries had planned to use generic fixed dose dolutegravir regimen, TLD (tenofovir/lamivudine/dolutegravir), which had received regulatory approval at the national level. With support from development partners Uganda had procured large quantities of TLD from the international market which the MOH staff noted would be sufficient in the medium term of the transition. South Africa had a relatively well-developed pharmaceutical industry compared with Uganda, and policymakers expected it to be able to meet domestic demand for TLD.

*“We already have large quantities of TLD that we purchased a while ago with the help of our partners. … I don’t think there will be problem with supply of TLD now, we have enough.”* (Uganda, MOH staff, P4)

*“I am optimistic that our local industry will be capable of producing the needed amount of TLD for the transition.”* (South Africa, MOH staff, P7)

Additionally, existing supply chain systems for ART and other medical supplies were deemed to be key opportunities to accelerate the transition in both countries.

#### Potential stockout of contraceptives

Participants reported frequent shortage of long-acting contraceptives in public health facilities in both countries as a potential barrier to the transition among women. They anticipated an increased demand for long-acting contraceptives following the rollout of dolutegravir-based regimen but noted no additional procurement arrangements had been made to increase supply. Most reported a lack of sufficient contraceptive options for HIV positive women who are often contraindicated for certain contraceptives.

*“nothing has been done on the contraceptive side … . I think the thinking is that it would be taken care of by what is currently available. We are likely to experience shortages … Most facilities in the hard to reach areas do not have enough family planning options; they only have condoms … . Some positive cannot use certain contraceptives, so we need to improve options.”* (Uganda, MOH staff, P12)

Part of the concern about contraceptive stockout was linked with funding shortage, which some participants in Uganda noted was partly due to some donors’ disinterest in contraception services.

*“The sector is already chronically underfunded... The problem is that some of our major donors are not interested in contraception. So you find that they will provide money for HIV but not for family planning.”* (Uganda, MOH staff, P12)

#### Weak stock management system

Regimen change requires an effective stock management system to minimise drug shortage and wastage. In South Africa, poor stock management at sub-national levels was reported to often create stockout of ARVs in health facilities. Participants emphasised the need for efficient systems to ensure proper forecasting and availability of ART and contraceptive commodities in health facilities.

*“It doesn't really matter whether there is enough drug at the depot it has to be at the facility. The problem with our system is that … there has been a lot of difficulty with understanding where the stock is. At the district level you think there should be enough stock, but everything is sitting in clinic A, whereas clinic B has no drug.”* (South Africa, MOH staff, P6)

Several MOH staff across both countries were also concerned that the WHO recommendation for a choice-based approach to ART among women of childbearing potential involving the use of either TLD or TLE/TEE (tenofovir/lamivudine/efavirenz; tenofovir/emtricitabine/efavirenz) would make it difficult to forecast actual commodity needs of the transition to ensure adequate supply.

*“If you are saying that you are going to give women a choice … , how do you quantify choice? How do you say that for every 5 women who come into the facility 5 of them will choose to have dolutegravir? it’s difficult to plan to ensure that the right commodities are available.”* (Uganda, Clinician, P6).

### Staff capacity and service delivery

#### Availability of ART service providers

Despite the above shortcomings, participants identified the availability of a network of ART service providers across the countries as a major opportunity for the transition. A key advantage noted was providers experience with previous ART regimen transitions. Whilst recognising their deficiencies, participants were optimistic that ART service providers could be empowered with improved infrastructure, equipment, personnel and training to accelerate the transition to dolutegravir.

*“We are lucky we already have our service providers; they are everywhere in the country, and they have experience with previous switch. All we need to do now is to train them in the new guidelines and give them resources and they will be ready to roll it out.”* (Uganda, MOH staff, P11).

#### Low capacity of health workers

Participants reported human resource crisis in the health sectors of both countries. Transition to dolutegravir-based regimen requires greater human resource capacity to deal with the extensive patient education, counselling and monitoring needs associated with treatment among women of childbearing potential. However, with existing acute shortage of health personnel and high client load in the health systems of South Africa and Uganda, there was concern about inadequate health workers for the transition and a potential worsening of ART service standard if the transition is implemented.

*“ … with our client load, low staffing norms, limited motivation of health workers, it will be hard for us to implement the counselling they [WHO] are asking for … . This is a big problem for the quality of ART service and the roll-out.”* (Uganda, Clinician, P10).

Further, participants recognised that most health workers involved in HIV care did not have the required skill set for dolutegravir-based treatment. Knowledge of current ART regimens and treatment protocols were deemed to be insufficient due to the potential risks of dolutegravir and the need for nuanced care for women of childbearing potential.

*“There hasn’t been training for about 11 or 12 years within the South African programme. We have just kind of been bashing along … . The switches we have had have not been particularly complicated. This one is going to be much more complicated.”* (South Africa, Researcher, P2).

Participants suggested the need for comprehensive training (rather than the usual orientation), including training in contraception for ART providers. They also suggested the need for dolutegravir-based treatment guidelines to be simplified to enable lower cadre health workers to implement.

#### Weak integration between HIV and contraception services

Effective contraception is recommended by the WHO for optimal dolutegravir use among women of childbearing potential. However, nearly all participants stated that family planning services in both countries were underfunded and deficient, characterised by frequent contraceptive shortage and lack of service in rural areas. Crucially, they identified weak integration between HIV and family planning services in both countries. South Africa was noted to have relatively better service compared with Uganda, although it was feared that the drawbacks could undermine the transition in both countries.

*“we need to make sure that contraception services are available in the ART clinics and that women are using them. That has been a weakness because in our health facilities the family planning clinic is usually on its own and ART clinic is on its own. … without integration how would you know if it is safe for woman to use dolutegravir?”* (Uganda, Clinician, P9).

### Community acceptability and uptake

#### Women’s desire for a regimen change

Nearly all participants across both countries identified that there was greater desire among women living with HIV for a regimen change due to the side effects of the current non-nucleoside reverse-transcriptase inhibitor-based regimens. They noted that this would promote greater community uptake of dolutegravir, being a better tolerated and more effective regimen, when it is rollout. However, there were concerns expressed among some MOH participants about how to meet community expectations with a higher demand.

*“I think it will be wowed by the community … . You know they have been complaining about the current drugs and are calling for something better. Now the problem is how to meet the high demand that would follow.”* (South Africa, MOH official, P7)

#### Limited knowledge about dolutegravir among women

Community awareness about dolutegravir was deemed to be essential for acceptability and uptake. However, not only was awareness about dolutegravir noted to be low in both countries, participants also reported of widespread misinformation in communities about dolutegravir due to publicity around the possible association with neural tube defects.

*“uptake will depend on how much people know. I mean it is easy for us here [in the city], but at the grassroots level … where they may not have access to all the information … they may say I am on my Efavirenz I am doing fine so I don’t need to change.”* (Uganda, Development Partner, P1).

High illiteracy among women was a key concern among participants, which was noted would hinder women’s ability to make optimal treatment choice. Many activists thus stressed the need for greater access to information about the risks and benefits of dolutegravir to engender better decision making on ART among women.

*“ … the African woman has been presented for so long that … . … ‘Oh, they will not be able to decide for themselves’. So, provide the choice with information so that they know and decide for themselves.”* (South Africa, Activist, P35)

#### Potential resistance to dolutegravir use in pregnancy

Several participants (mostly activists and development partners) were concerned that the potential risk of neural tube defects would discourage vulnerable groups with limited information from using dolutegravir. They feared that most men would prevent their spouses from using dolutegravir-based regimens due the potential risks in pregnancy.

*“People are taking the neural tube defects news serious and because of that they will reject dolutegravir. … you know in our culture pregnancy and children are very important. … even if the woman likes it, the man will tell her not to use it.”* (Uganda, Activist, P7)

### Post-market surveillance

#### Limited systems for pharmacovigilance in pregnancy

There was widespread recognition of the need for the rollout of dolutegravir-based regimens to be accompanied by intensive pharmacovigilance in pregnancy due to the potential teratogenicity of the drug. However, existing pharmacovigilance systems in South Africa and Uganda were noted to be inadequate for effective toxicity monitoring in pregnancy. Both countries relied heavily on general pharmacovigilance (spontaneous reporting system) for ART and constrained by severe underreporting, low funding and poor coordination.

*“our pharmacovigilance system in this country is not efficient enough to follow up the roll out of these drugs [ARVs] in pregnant women. … we rely mainly on spontaneous reporting, even for ART.”* (Uganda, Regulator, P11).

South Africa had a limited pregnancy pharmacovigilance system for ART in the form of two provincial level pregnancy registries. However, participants noted that the registries were too expensive to scale up nationally, lacked effective mechanisms for analyses, interpretation and use of the data, and did not cover postpartum risks. Some regulators in Uganda expressed the desire for a pregnancy registry to support the rollout of dolutegravir in the country but noted they lacked financial resources and technical expertise to establish one.

*“I am always preaching for pregnancy registry especially now that DTG is coming. But whenever I bring it up [in meetings with the ministry] they will say there is no money to do it. … they will say it is our remit, but they will not provide the funds. … we need help like technical expertise”* (Uganda, Regulator, P11).

## Discussion

This study explored the health system implications of introducing dolutegravir-based regimens among women of childbearing potential in two resource-limited settings in sub-Saharan Africa. Whilst transition to a dolutegravir-based first line regimen is fraught with challenges, it presents opportunities for health system strengthening. Challenges identified across the two countries include potential gender inequity, limited data on community preferences and safety of dolutegravir use in pregnancy, weak stock management system and potential stock out of effective contraceptives, limited health worker capacity, weak integration between HIV and contraception services, and limited pharmacovigilance in pregnancy. Studies from elsewhere indicate that these are likely to be relevant to a large number of LMIC contexts where HIV is endemic [3, 29]. The current political will for transition (both from national governments and international development partners) coupled with existing health system resources provide opportunities for tackling the challenges particularly in contraceptive services and pregnancy pharmacovigilance.

Our findings revealed a tension between the rapid pace of transition and the weak health system preparedness of the study countries. The complexity and scale of the transition require comprehensive planning and preparation, yet there was immense pressure from donors and civil society organisations for health systems to transition quickly and to allow women of childbearing potential to access dolutegravir-based regimens despite health systems being ill-prepared to implement the change in regimen. This resulted in the development of tentative guidelines with no robust evidence to support critical decisions. Future transition would merit a gradual approach that allows for all the necessary institutional and individual capacities to be strengthened and for essential evidence to be collated to inform the transition process.

The availability of adequate robust data on dolutegravir use in pregnancy is central to both policy and clinical decisions in transition. Important data gaps were the safety of dolutegravir use in pregnancy (especially at periconception and early pregnancy), switching between regimens during pregnancy, rollout implementation and community values and preference on dolutegravir-based treatment. Previous studies have reported gaps in evidence on dolutegravir use among HIV-TB coinfected people and among people who have not been evaluated for drug resistance before starting treatment [10]. Whilst both observational and clinical trial data may be useful in addressing these gaps, our study found that health system stakeholders are most persuaded by clinical trial data. Data from the Tsepamo study and ongoing trials on dolutegravir such as DolPHIN-2 may address some of these gaps. Operational data and community feedback from early adopter countries are also needed to facilitate a successful transition.

Establishing an effective national pharmacovigilance system for ART is critical for toxicity monitoring and pregnancy safety surveillance. The results show that stakeholders are most concerned about the risks of dolutegravir exposure in pregnancy and lactation, suggesting the need for pharmacovigilance to focus on both areas. Spontaneous reporting systems common in most LMICs, with their low and biased reporting, provide limited data to address the surveillance needs [30]. The WHO pregnancy exposure registry has over the years provided the blueprint for pregnancy pharmacovigilance in many LMICs, including South Africa, and is useful for delivering comprehensive data on maternal health and ARV exposure risks in pregnancy [31]. The high uptake of antenatal care [31] and existing clinical programmes (e.g. antenatal care and ART) in many LMICs (including South Africa and Uganda) are useful opportunities for creating a pregnancy registry. However, as observed in the interviews in South Africa and other studies [30], such pregnancy registries are resource intensive and fail to address risks associated with exposure in lactation. Additionally, establishing pregnancy pharmacovigilance systems may be constrained by limited human resource capacity to monitor birth outcomes and toxicities, lack of diagnostic equipment for case detection, low staff and patient motivation to engage, limited access to antenatal and postnatal services in rural areas, and poor reporting systems [32]. Consequently, health systems in LMICs may need a more pragmatic and cost-effective pregnancy pharmacovigilance system that would yield the necessary treatment and comparison data on dolutegravir use in pregnancy and lactation to ascertain both long- and short -term toxicities. The high turnover of ART regimen means that such a system should be amenable to different ARV drugs and integrated into the healthcare system to ensure its long-term sustainability. At policy level, it is essential to strengthen the legislative and regulatory framework and financial support for ART pharmacovigilance. Limitations in these account for much of the weakness in the ART pharmacovigilance systems in most LMICs [32].

The WHO recommendation to make dolutegravir accessible to all women regardless of reproductive potential or plans [8], which has now been adopted in Uganda (although not included in original guidelines [13]), would significantly alleviate equity concerns around dolutegravir use in women [33]. However, community perceptions of the teratogenicity of dolutegravir are unlikely to completely disappear overnight and may continue to undermine uptake among women. Achieving universal coverage of dolutegravir-based treatment among women requires greater community awareness and education. Such demand generation efforts should be embedded in a comprehensive community engagement approach that targets both HIV positive and negative persons to raise awareness and collate community feedback. Engaging with men would be critical to enhancing dolutegravir use among women due to the widespread patriarchal norms in these societies [34].

Access to effective contraception continues to be critical to dolutegravir transition. The WHO 2019 guidelines, adopted by both South Africa and Uganda, stipulate that women are informed about the risks associated with dolutegravir use in pregnancy combined with an offer of choice of contraception [8]. Evidence suggest that low access to and uptake of effective contraceptive methods among women living with HIV in LMICs, linked with concerns about health risks relating to side effects, contraceptive failure arising from drug-drug interactions with ARVs, and lack of contraception services [35, 36] . There is the need for an improvement in the availability of these contraception services combined with health care worker and community-based interventions to promote positive attitudes toward contraception among women living with HIV. Integration of contraception and ART services, as noted by the stakeholders, is essential to effective transition. Although potentially feasible and cost-efficient to implement in resource-limited settings [37], integration was reported to be weak in both South Africa and Uganda. Strengthening this would require action at policy and service delivery levels, including common guidelines and funding for both services, increasing staffing for and training in contraception services, reorienting service delivery to create one-stop centres for accessing both services, and improving referral systems [38].

The choice-based approach to dolutegravir use among women of childbearing age recommended by the WHO [2, 8] and adopted by South African [14] and Ugandan [13] ART guidelines, is necessary to promote women’s autonomy and ART uptake and adherence, but may undermine treatment harmonisation by requiring differentiated service provision. Similar to previous observations [39], our findings suggest that actual implementation of informed choice may be constrained by widespread patriarchy, medical paternalism and high illiteracy among women. Promoting informed choice at the community level requires careful attention to cultural sensitivities of empowering women to make their own choices, while seeking to reorient the decision-making relationship between women and their partners. Health workers should also be enabled to support patients with decision-making through the provision of adequate training and information. The complexity of balancing the risks of dolutegravir in pregnancy against the limited experience on choice-based ART service delivery, suggests the need for further support on how to communicate these risks and choices. Further, improving the availability of a range of contraception options and alternative ART regimens (e.g. efavirenz-based regimens) would be key to enhancing choice and enabling women to access their preferred treatments.

Our findings present several implications for key health systems stakeholders. Policymakers and programmers need to strengthen supply chain capacity and internal stock management systems to ensure continuous availability of ART and contraception commodities. The pricing agreement on dolutegravir negotiated by UNAIDS and other partners could be leveraged to improve the availability of generic versions of dolutegravir-based regimens [40]. They should also revise ART guidelines and simplify these to make them assessible to lower cadre health workers, including ART prescribers learning about how to administer contraception services in an integrated HIV – contraceptive service delivery model. Donors and development partners need to grant the opportunity (including financial and technical support) for health systems to adequately strengthen their capacities and collate the necessary evidence to inform the transition.

### Limitations

The interviews were conducted at a time when the two study countries had not started implementing the transition to dolutegravir-based regimens. Therefore, the findings are based mainly on respondents’ perceptions of anticipated challenges and outcomes rather than data from the actual implementation of the transition. However, responses were mainly informed by participants’ depth of knowledge of the health systems and experience of previous ART transitions, which would have strengthened the validity of the findings. The qualitative design and small sample size of the study may have limited the generalisability of the findings. Nevertheless, our use of purposive sampling approach ensured that we were able to capture views that were typical of the groups that the respondents represented, and we reached thematic saturation. Due to the exploratory design of the study we were only able to provide a brief account of the issues related to the transition and could not delve into greater detail for each of them: each of the discussed topics could merit a separate detailed analysis within specific health system contexts. Some of the researchers (LM, ML, AK) were members of the ART guidelines group of their respective countries and provided guidance in identifying potential participants for the study which may have influenced participants selection and the study data. To minimise bias, their input to the study was limited to advising on the study design and discussing and reviewing draft results; they neither participated as interviewees or interviewers nor directly analysed the transcripts.

## Conclusion

Our findings demonstrate the complexity of transitioning to dolutegravir-based regimens among women of childbearing potential in low-resource context. We observed that despite numerous challenges to transition, driven in part by the bottlenecks of the weak health systems of LMICs and the rapid pace of change, that transition provides potential opportunities for strengthening contraceptive and pregnancy pharmacovigilance. The challenges of transition are far-reaching and pervade different aspects of the health system, suggesting the need for a multi-sectoral effort to facilitate a successful rollout. This should include extensive community engagement before and during the transition, as well as individual and institutional capacity building, including comprehensive staff training and improvement in counselling and contraception services, with focus on the strengthening of pregnancy pharmacovigilance. Further observation and trial data are required to better understand the safety of dolutegravir and facilitate community uptake and adoption by health policy makers. In future, health systems should develop mechanisms to record experiences from previous transitions, in order to inform future transitions and reduce repeated mistakes. The push for transition to new ART drugs needs to recognise the constraints of health systems in LMICs and to allow for adequate planning and preparation prior to transition.

## Supplementary information

**Additional file 1.** Topic guide for interview with health system stakeholders.

## Data Availability

Data analysed for this manuscript are available from the corresponding author on reasonable request**.**

## Abbreviations

DolPHIN: Dolutegravir in pregnant HIV mothers and neonates
HIV: Human immunodeficiency virus
LMIC: Low- and middle-income country
MOH: Ministry of health
TEE: Tenofovir, emtricitabine, efavirenz
TLD: Tenofovir, lamivudine, dolutegravir
TLE: Tenofovir, lamivudine, efavirenz
WHO: World health organisation
TWG: Technical working group
