# Supplementary material for: Engendering health systems in response to national rollout of dolutegravir-based regimens among women of childbearing potential: a qualitative study with stakeholders in South Africa and Uganda
Source: BMC Health Serv Res. 2020 Aug 1;20:705. doi: 10.1186/s12913-020-05580-0 (PMC7395396; doi:10.1186/s12913-020-05580-0)
Supplement: Supplementary file 1 — Additional file 1. Topic guide for interview with health system stakeholders. [file 12913_2020_5580_MOESM1_ESM.doc]

**Topic guide for interview with health system stakeholders**

1. Please tell me a little about yourself: where you work, your role, how long you have worked in that role, etc

- Probe role in HIV policy, programmes and guidelines development.

1. Tell me about the discussions on the transition to dolutegravir, and how you may have been involved.
2. What decisions have been taken on the transition so far?

- PROBE: would women be allowed to use dolutegravir? How? Why?
- PROBE: would women who present late for antenatal care with untreated HIV be allowed to use dolutegravir?
- PROBE: what safeguarding measures will be put in place to protect women?
- PROBE: has the Botswana findings (Tsepamo study) had any impact on decisions about the transition, if so how? .

1. Are you satisfied with the decision on how dolutegravir will be rolled out in this country? How?

- PROBE: if not satisfied, what changes would you propose?

1. Tell me about how the development of the guidelines for the transition in progressing

- PROBE: what has been the main challenges? How are you dealing with these?

1. What arrangements has the government made so far for the transition?

- PROBE: timelines for transition, funding, training, drug procurement, guidelines development, etc.
- PROBE: Is the health system ready for the transition? how?

1. How might the transition impact health service delivery?

- PROBE: what preparations has the government made regarding health service delivery?
- PROBE: what implications might the transition have on sexual and reproductive health services?

1. What other challenges do you foresee affecting the transition in future?

- PROBE: challenges with transition among women
- PROBE: how the challenges might be mitigated

1. Are there any lessons from the previous transition (e.g. Nevirapine to Efavirenz) that should inform the current transition? how?
2. Tell me about the pharmacovigilance system in this country, and how it might be used in the transition.

- PROBE: systems for pregnancy pharmacovigilance
- PROBE: challenges with pregnancy pharmacovigilance
- PROBE: measures for improving pregnancy pharmacovigilance

1. What key opportunities exist in the health system that you think should be harnessed to facilitate the transition?
2. What are the next steps (for your organisation) regarding the roll out of dolutegravir?
3. Is there anything else you expected us to discuss that we did not?
4. Are there any other persons you would recommend for us to interview on this topic?
